# Associations between coronary heart disease and risk of cognitive impairment: A meta‐analysis

**DOI:** 10.1002/brb3.2108

**Published:** 2021-03-20

**Authors:** Xuan Liang, Yilin Huang, Xu Han

**Affiliations:** ^1^ Nanjing University of Chinese Medicine Nanjing China; ^2^ Affiliated of Hospital of Nanjing University of Chinese Medicine Nanjing China

**Keywords:** Alzheimer's disease, coronary heart disease, dementia, meta‐analysis

## Abstract

**Background:**

Several studies have demonstrated that coronary heart disease (CHD) is a high risk factor for cognitive impairment, whereas other studies showed that there was no association between cognitive impairment and CHD. The relationship between CHD and cognitive impairment is still unclear based on these conflicting results. Thus, it is of importance to evaluate the association between CHD and cognitive impairment. The present study made a meta‐analysis to explore the association between CHD and risk of cognitive impairment.

**Methods:**

Articles exploring the association between CHD and cognitive impairment and published before November 2020 were searched in the following databases: PubMed, Web of Science, Medline, EMBASE, and Google Scholar. We used STATA 12.0 software to compute the relative risks (RRs), odds ratios (ORs), or hazard ratios (HRs) and 95% confidence intervals (CIs).

**Results:**

The meta‐analysis showed a positive association between CHD and risk of all‐cause cognitive impairment with a random effects model (RR = 1.27, 95% CI 1.18 to 1.36, I^2^ = 82.8%, *p* < .001). Additionally, the study showed a positive association between myocardial infraction (MI) and risk of all‐cause cognitive impairment with a random effects model (RR = 1.49, 95% CI 1.20 to 1.84, I^2^ = 76.0%, *p* < .001). However, no significant association was detected between angina pectoris (AP) and risk of all‐cause cognitive impairment with a random effects model (RR = 1.23, 95% CI 0.95 to 1.58, I^2^ = 79.1%, *p* < .001). Subgroup studies also showed that CHD patients are at higher risk for vascular dementia (VD), but not Alzheimer's disease (AD) (VD: RR = 1.34, 95% CI: 1.28–1.39; AD: RR = 0.99, 95% CI: 0.92–1.07).

**Conclusion:**

In a word, CHD was significantly associated with an increased risk of developing cognitive impairment.

## Graphical abstract

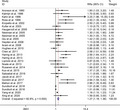

## 1 INTRODUCTION

Coronary heart disease (CHD), one of the major cardiovascular diseases, is the leading cause of death and disability affecting the global human population (Ang & Chan, 2016). In the United States, CHD accounts for about 25% of all deaths annually (Brown et al., 2020). CHD is closely related to atherosclerosis, and the development of atherosclerotic plaques in the vessel walls of the coronary arteries supplying the heart decreased the myocardial perfusion (Potz et al., 2017). CHD is a range of clinical disorders manifested as stable and unstable angina, myocardial infraction (MI), or sudden cardiac death (Álvarez‐Álvarez et al., 2017; Libby & Theroux, 2005). There are many risk factors of developing CHD, including obesity, excessive salt intake, excessive drinking, and smoking (Shao et al., 2020).

Cognitive impairment is common in the elderly and is characterized by deterioration of memory, attention, and cognitive function beyond what is expected based on age and educational level (Eshkoor et al., 2015). Dementia is a state that acquired cognitive impairment has been serious enough to affect normal social and/or occupational functioning (de Souza‐Talarico et al., 2016). Studies reported that the prevalence of dementia floats between 5% and 7% worldwide and is higher in the developing countries (de Souza‐Talarico et al., 2016). Cognitive impairment and dementia will place huge individuals, societal and financial burdens, given that the aging population globally is increasing (Dye et al., 2017). And more importantly, there are no effective treatments proven to stop or slow the progression of mild cognitive impairment to dementia.

Previous studies provided inconsistent results regarding the association between CHD and risk of cognitive impairment. Several studies have demonstrated that CHD is a high‐risk factor for cognitive impairment (Gondim et al., 2017; Mahon et al., 2017), whereas other studies showed that there was no association between cognitive impairment and CHD (Xing et al., 2020; Yang et al., 2020). The relationship between CHD and cognitive impairment is still unclear based on these conflicting results. Thus, it is of importance to evaluate the association between CHD and cognitive impairment. The present study made a meta‐analysis to explore the association between CHD and risk of cognitive impairment.

## 2 Methods

The present study was conducted based on the Preferred Reporting Items for Systematic reviews and Meta‐Analysis (PRISMA) guideline (Moher et al., 2009).

### 2.1 Search strategy and selection criteria

Two researchers (Xuan Liang and Yilin Huang) searched for articles published before November 2020 in the following databases: PubMed, Web of Science, Medline, EMBASE, and Google Scholar. Search terms were used as follows: (‘coronary heart disease’ OR ‘myocardial infarction’ OR ‘angina pectoris’) AND (‘dementia’ OR ‘cognitive deficits’ OR ‘cognitive dysfunction’ OR ‘cognitive impairment’ OR ‘Alzheimer's disease’). Selection criteria included the following: (1) Relative risks (RRs), odds ratios (ORs), or hazard ratios (HRs) and 95% confidence intervals (CIs) associated with CHD and risk of cognitive impairment could be reported or calculated from included studies; (2) we eliminated meta‐analyses, reviews, and case‐reports.

### 2.2 Data extraction

The following data were extracted from included studies. These data included: Author, publication year, study type, study location, sample sizes, information of participants (age and gender), CHD type, median follow‐up time, type and cases of cognitive impairment, adjustment variables, and results. In addition, an Excel file was used to abstract these data.

### 2.3 Meta‐analysis

STATA 12.0 software was used to compute the results. Heterogeneities between studies were evaluated with the Q test and I^2^. With invariably high heterogeneity (*p* value for *Q* test ≤ .05 and I^2^ ≥ 50%), random effects models were used as pooling methods; with invariably low heterogeneity (*p* value for *Q* test > .05 and I^2^ < 50%), fixed effects models were used. Sensitivity analysis was used to evaluate the stabilization of the study. In addition, Begg's test, Egger's test, and funnel plot were used to evaluate publication bias.

## 3 RESULTS

### 3.1 Study selection and characteristics

Figure S1 illustrated the selection results. Table S1 showed study characteristics and results of included studies. A total of 28 studies (Aronson et al., 1990; Chen et al., 2011; Gondim et al., 2017; Haring et al., 2013; Hayden et al., 2006; Hughes et al., 2010; Ikram et al., 2008; Jacob et al., 2017; Kahn et al., 1996; Kalmijn et al., 1996; Kivipelto et al., 2002; Kuller et al., 2003; Kuo et al., 2015; Li et al., 2011; Lipnicki et al., 2013; Mahon et al., 2017; Newman et al., 2005; Noale et al., 2013; Qiu et al., 2005; Ricotti et al., 2016; Ross et al., 1999; Rusanen et al., 2014; Satizabal et al., 2016; Solfrizzi et al., 2004; Sundbøll et al., 2018; Verhaegen et al., 2003; Xing et al., 2020; Yang et al., 2020) (including 1,397,314 participants) explored the association between CHD and risk of cognitive impairment.

### 3.2 Meta‐analysis results

**FIGURE 1:**
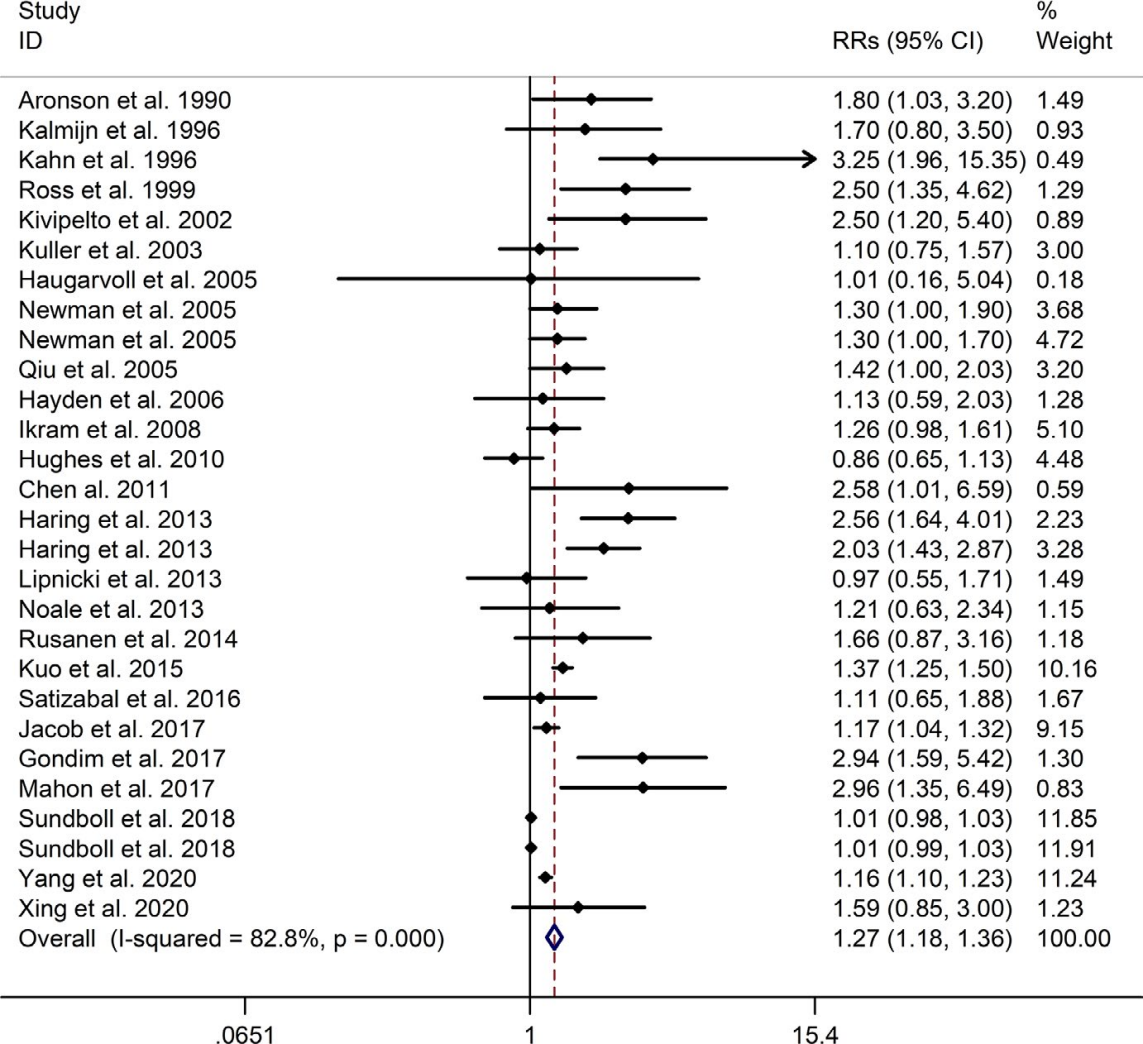
Forest plot regarding association between CHD and risk of all‐cause cognitive impairment. Abbreviations: CHD, coronary heart disease; OR, odds ratio; RR, relative risk

**FIGURE 2:**
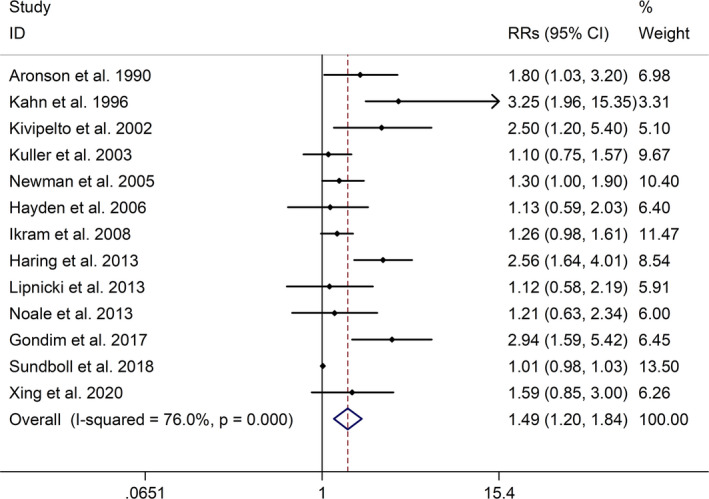
Forest plot regarding association between MI and risk of all‐cause cognitive impairment. Abbreviations: MI, myocardial infraction; OR, odds ratio; RR, relative risks

**FIGURE 3:**
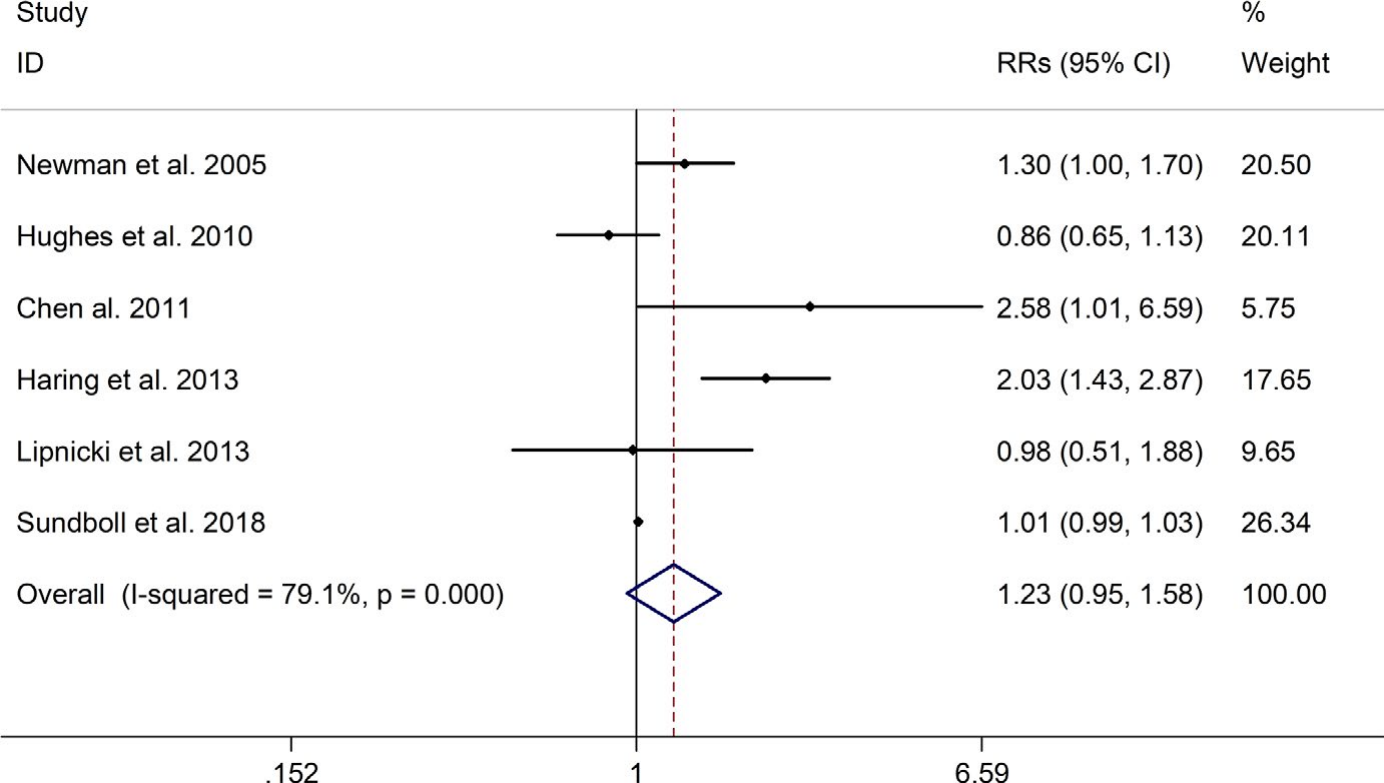
Forest plot regarding association between AP and risk of all‐cause cognitive impairment. Abbreviations: AP, angina pectoris; OR, odds ratio; RR, relative risks

**FIGURE 4:**
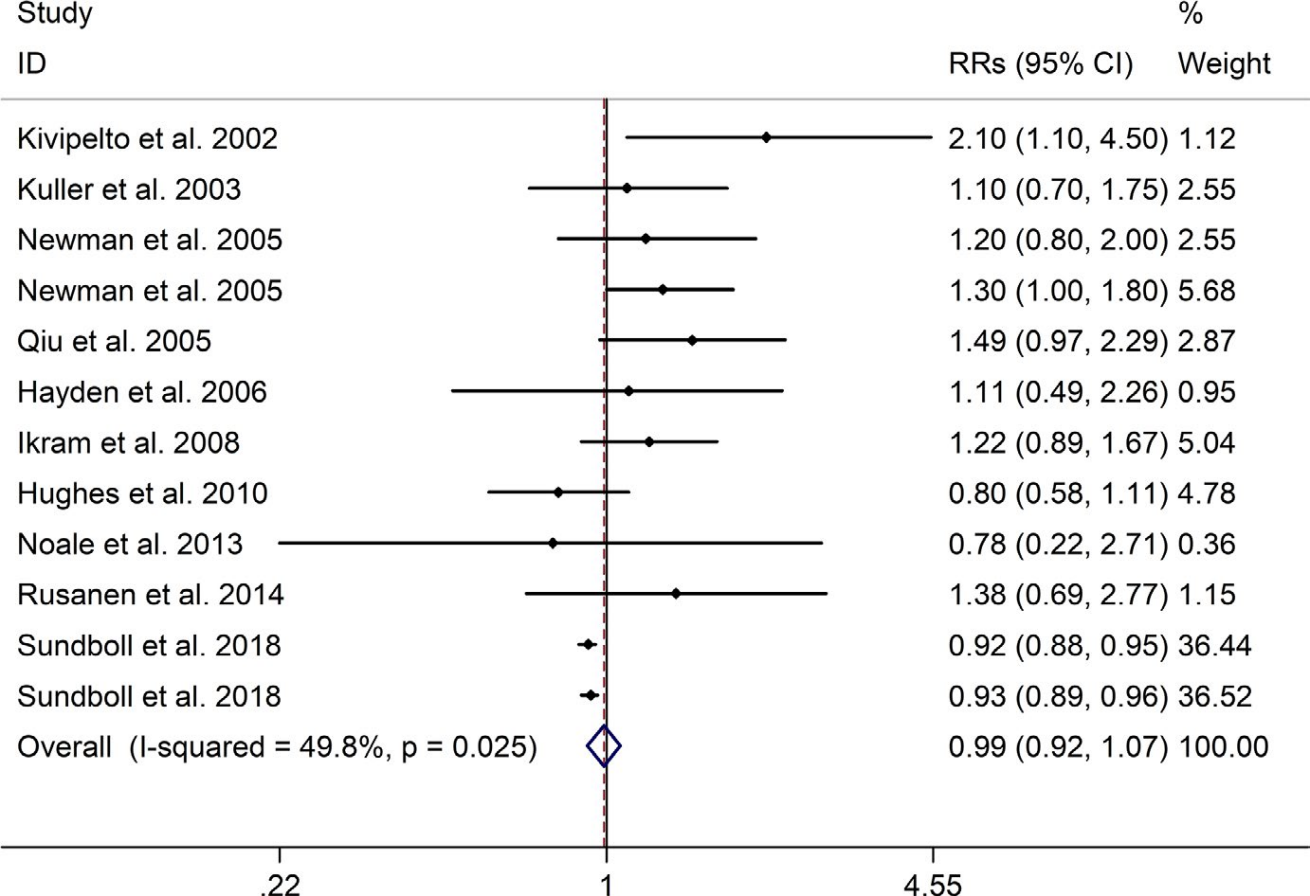
Forest plot regarding association between CHD and risk of AD. Abbreviations: AD, Alzheimer's disease; CHD, coronary heart disease; OR, odds ratio; RR, relative risks

**FIGURE 5:**
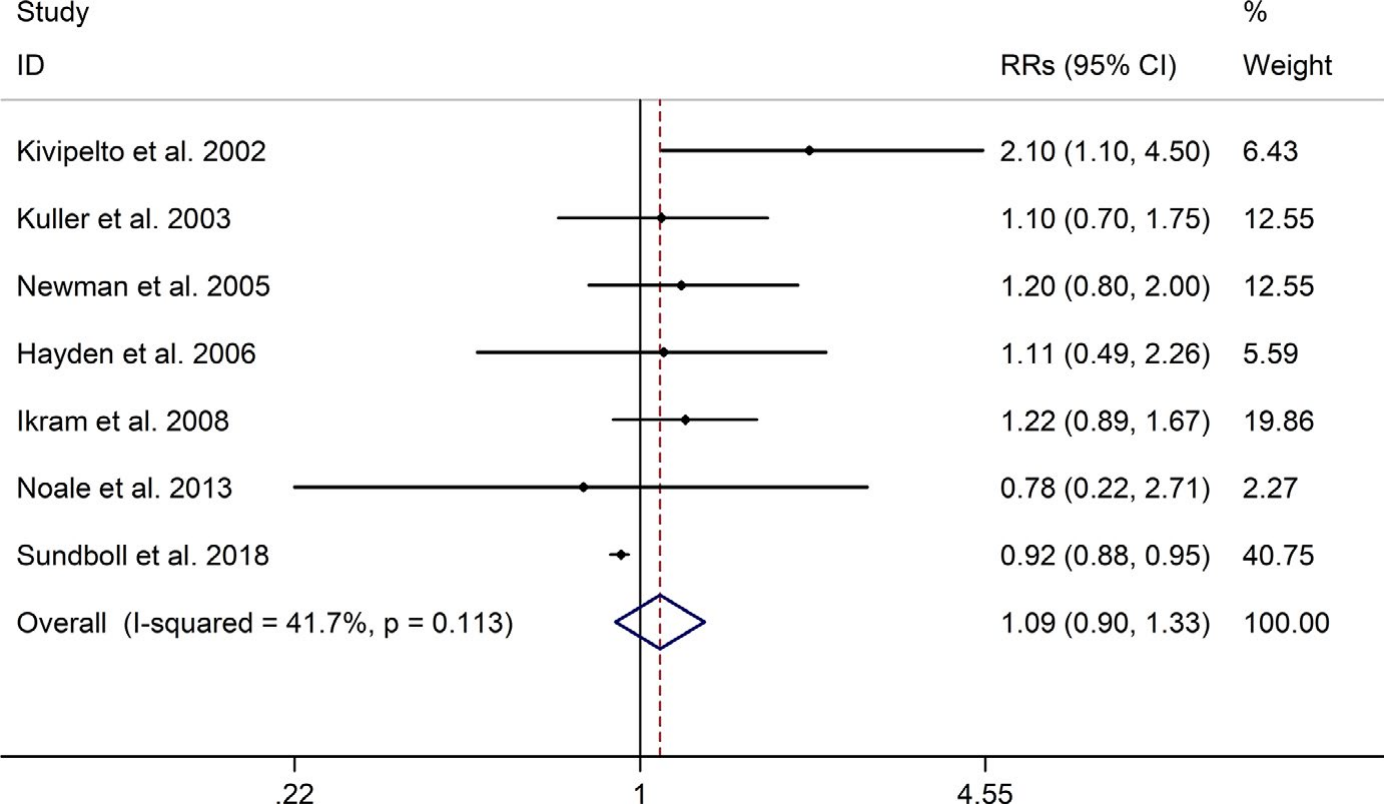
Forest plot regarding association between MI and risk of AD. Abbreviations: AD, Alzheimer's disease; MI, myocardial infraction; OR, odds ratio; RR, relative risks

**FIGURE 6:**
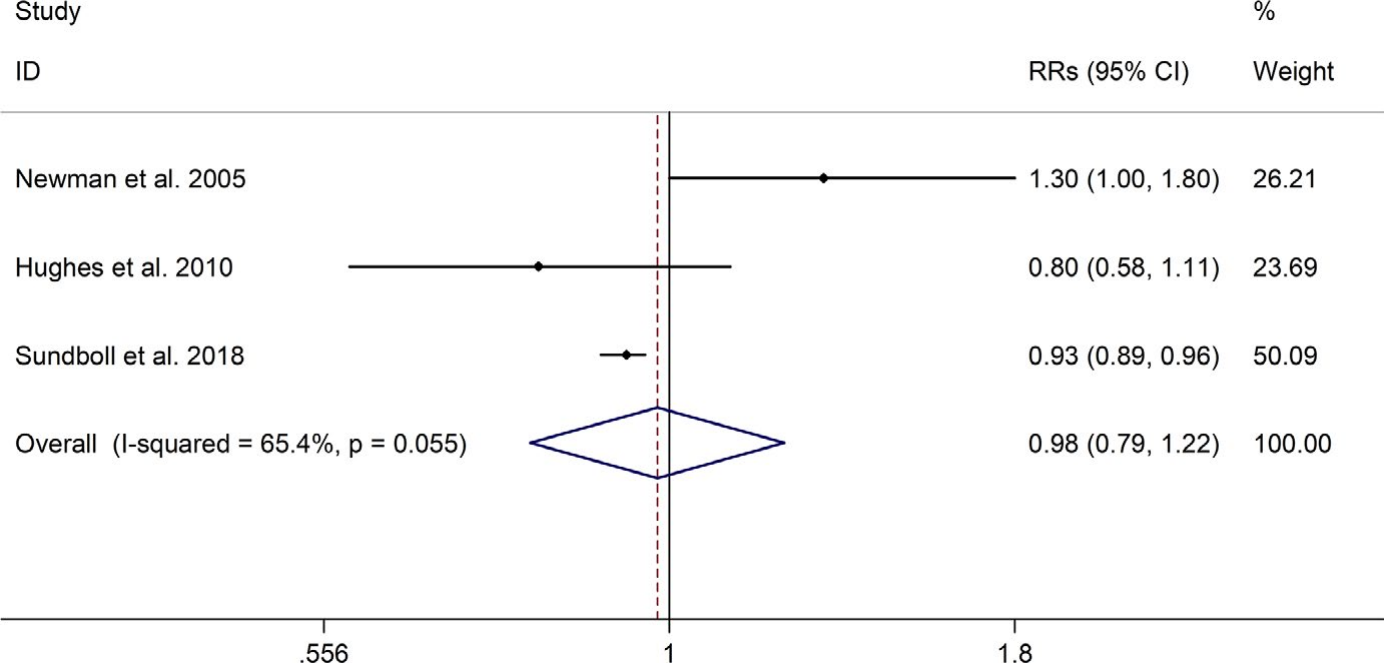
Forest plot regarding association between AP and risk of AD. Abbreviations: AD, Alzheimer's disease; AP, angina pectoris; OR, odds ratio; RR, relative risks

**FIGURE 7:**
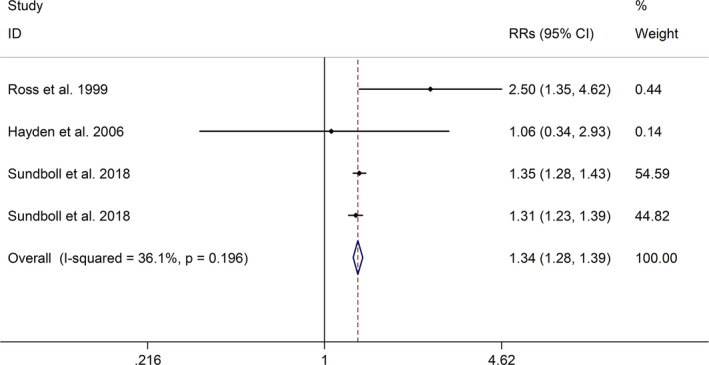
Forest plot regarding association between CHD and risk of VD. Abbreviations: CHD, coronary heart disease; OR, odds ratio; RR, relative risks; VD, vascular dementia

The meta‐analysis showed a positive association between CHD and risk of all‐cause cognitive impairment with a random effects model (RR = 1.27, 95% CI 1.18 to 1.36, I^2^ = 82.8%, *p* < .001, Figure 1). Additionally, the study showed a positive association between MI and risk of all‐cause cognitive impairment with a random effects model (RR = 1.49, 95% CI 1.20 to 1.84, I^2^ = 76.0%, *p* < .001, Figure 2). However, no significant association was detected between angina pectoris (AP) and risk of all‐cause cognitive impairment with a random effects model (RR = 1.23, 95% CI 0.95 to 1.58, I^2^ = 79.1%, *p* < .001, Figure 3). No significant association was showed between CHD and risk of AD with a random effects model (RR = 0.99, 95% CI 0.92 to 1.07, I^2^ = 49.8%, *p* = .025, Figure 4). In addition, no significant association was showed between MI and risk of Alzheimer's disease (AD) with a fixed effects model (RR = 1.09, 95% CI 0.90 to 1.33, I^2^ = 41.7%, *p* = .113, Figure 5). No significant association was showed between AP and risk of AD with a random effects model (RR = 0.98, 95% CI 0.79 to 1.22, I^2^ = 65.4%, *p* = .055, Figure 6). However, a positive association was detected between CHD and risk of vascular dementia (VD) with a fixed effects model (RR = 1.34, 95% CI 1.28 to 1.39, I^2^ = 36.1%, *p* = .196, Figure 7). Sensitivity analyses showed no changes in the direction of effect when any one study was excluded for the studies in all meta‐analyses (Figures S2 and S3). Begg's test, Egger's tests, and funnel plots showed significant risks of publication bias for studies on associations between CHD and risk of all‐cause cognitive impairment, MI and risk of all‐cause cognitive impairment, CHD and AD, MI and AD (CHD and risk of all‐cause cognitive impairment: Begg's test: *p* = .06; Egger's test: *p* < .001; Figure S4. A; MI and risk of all‐cause cognitive impairment: Begg's test: *p* = .31; Egger's test: *p* = .001; Figure S4. B; CHD and AD: Begg's test: *p* = .07; Egger's test: *p* = .01; Figure S4. D; MI and AD: Begg's test: *p* = .30; Egger's test: *p* = .047; Figure S5. A). However, Begg's test, Egger's tests, and funnel plots showed no significant risks of publication bias for studies on associations between AP and risk of all‐cause cognitive impairment, AP and AD, CHD and VD (AP and risk of all‐cause cognitive impairment: Begg's test: *p* = .260; Egger's test: *p* = .214; Figure S4. C; AP and AD: Begg's test: *p* = .602; Egger's test: *p* = .755; Figure S5. B; CHD and VD: Begg's test: *p* = .34; Egger's test: *p* = .519; Figure S5C).

## 4 DISCUSSION

A total of 28 studies, investigating the association between CHD and cognitive impairment with 1,397,314 participants, were included in this meta‐analysis. The results showed that CHD is strongly related with the risk of all‐cause cognitive impairment (RR = 1.27, 95% CI: 1.18–1.36). And subgroup studies showed that there is a positive association between MI and cognitive impairment, but no association between AP and all‐cause cognitive impairment (MI: RR = 1.49, 95% CI: 1.20–1.84; AP: RR = 0.99, 95% CI: 0.92–1.07). Subgroup studies also showed that CHD patients are at higher risk for VD, not AD (VD: RR = 1.34, 95% CI: 1.28–1.39; AD: RR = 0.99, 95% CI: 0.92–1.07). And our study was partly consistent with a previous study published in 2017 (Deckers et al., 2017). Deckers et al. reported that patients with CHD have a 45% increased risk of cognitive impairment or dementia based on included prospective cohort studies, and according to included cross‐sectional studies there were no associations between CHD and cognitive impairment or dementia (Cohort studies: OR = 1.45, 95%CI: 1.21–1.74; Cross‐sectional studies: OR = 1.23, 95%CI = 0.76–1.97) (Deckers et al., 2017).

The potential mechanisms of high risk of cognitive impairment among CHD patients are still unknown. Previous studies showed that several common risk factors in elderly population, including low physical activity, diabetes mellitus, hypertension, are associated with risk of both CHD and cognitive impairment (Booth et al., 2012; Escobar, 2002; Naito & Miyauchi, 2017; Santisteban & Iadecola, 2018; Yuan & Wang, 2017). Thus, these accepted cardiovascular risk factors contribute to the increased risk of cognitive impairment and dementia. In addition, multiple comorbid cardiovascular risk factors accumulate the risk of dementia (Whitmer et al., 2005). Bleckwenn et al. showed that the course of cognitive decline is influenced by CHD in older people with a recent diagnosis of dementia (Bleckwenn et al., 2017). Atrial fibrillation (AF) is the most common cardiac arrhythmia, and the prevalence of AF among the CHD patients is estimated from 0.2% to 5%. Arrhythmia is common after cardiac surgeries and accounts for about 20% to 40% of the patients after coronary artery bypass graft (CABG) surgery (Michniewicz et al., 2018). AF has been considered as a highly related risk factor of cognitive impairment (Sepehri Shamloo et al., 2020). Besides, CABG surgery has been related to cognitive impairment, and Greaves reported that approximately 40% of patients were diagnosed with cognitive impairment between 1 and 5 years postoperatively (Greaves et al., 2019). Atherosclerosis progression is intimately linked with CHD and affects the integrity and function of cerebral vessels resulting in the impairment of cerebral blood flow and cerebrovascular dysfunction (Shabir et al., 2018), which may partly explain the association between CHD and VD. Some mechanisms such as micro‐emboli and/or decreased cardiac output to the brain, the release of inflammatory molecules may also paly role in the cognitive impairment (Abete et al., 2014; Corona et al., 2012).

This meta‐analysis showed the significant association between CHD and cognitive impairment. Both CHD and cognitive impairment are common in the aged, and we hope our study contribute to a better understanding of the association between cardiovascular and cognitive function. However, there are some limitations in this study. First, due to the restriction of the source of data, risk estimates for cognitive impairment could be obtained from all studies, which may lead to selection bias. Second, certain publication bias was found in this study, some results might be distorted. And despite the use of fully adjusted models, residual confounding in primary studies could affect the results. Therefore, it is emphasized that additional high‐quality clinical studies and biological mechanism research are needed to assess the association between CHD and cognitive impairment. Finally, regarding the association between MI/AP and risk of dementia or CI, there were a limited number of studies, potentially limiting statistical power. More large‐scale studies might be performed to explore the association between MI/AP and risk of dementia or CI.

## 5 CONCLUSIONS

In a word, CHD was significantly associated with an increased risk of developing cognitive impairment. However, it is emphasized that additional high‐quality clinical studies and biological mechanism research are needed to assess the association between CHD and cognitive impairment.

## CONFLICTS OF INTEREST

On behalf of all authors, the corresponding author states that there is no conflict of interest related to this study.

## AUTHOR CONTRIBUTION

Xuan Liang and Yilin Huang were in charge of the writing of the paper, the performance of the research and data analysis. Xu Han was in charge of research design and data analysis.

## FUNDING

This study was supported by the Natural Science Foundation of Jiangsu Province (No.BK20181505).

## ETHICAL STATEMENT

The study was a meta‐analysis. Thus, ethical statement is not applicable.

### Peer Review

The peer review history for this article is available at https://publons.com/publon/10.1002/brb3.2108.

## Supporting information

Fig S1

Fig S2

Fig S3

Fig S4

Fig S5

Table S1

Supplementary Material
