# Supplementary figures and images for: Associations between coronary heart disease and risk of cognitive impairment: A meta‐analysis
Source: Brain Behav. 2021 Mar 20;11(5):e02108. doi: 10.1002/brb3.2108 (PMC8119850; doi:10.1002/brb3.2108)

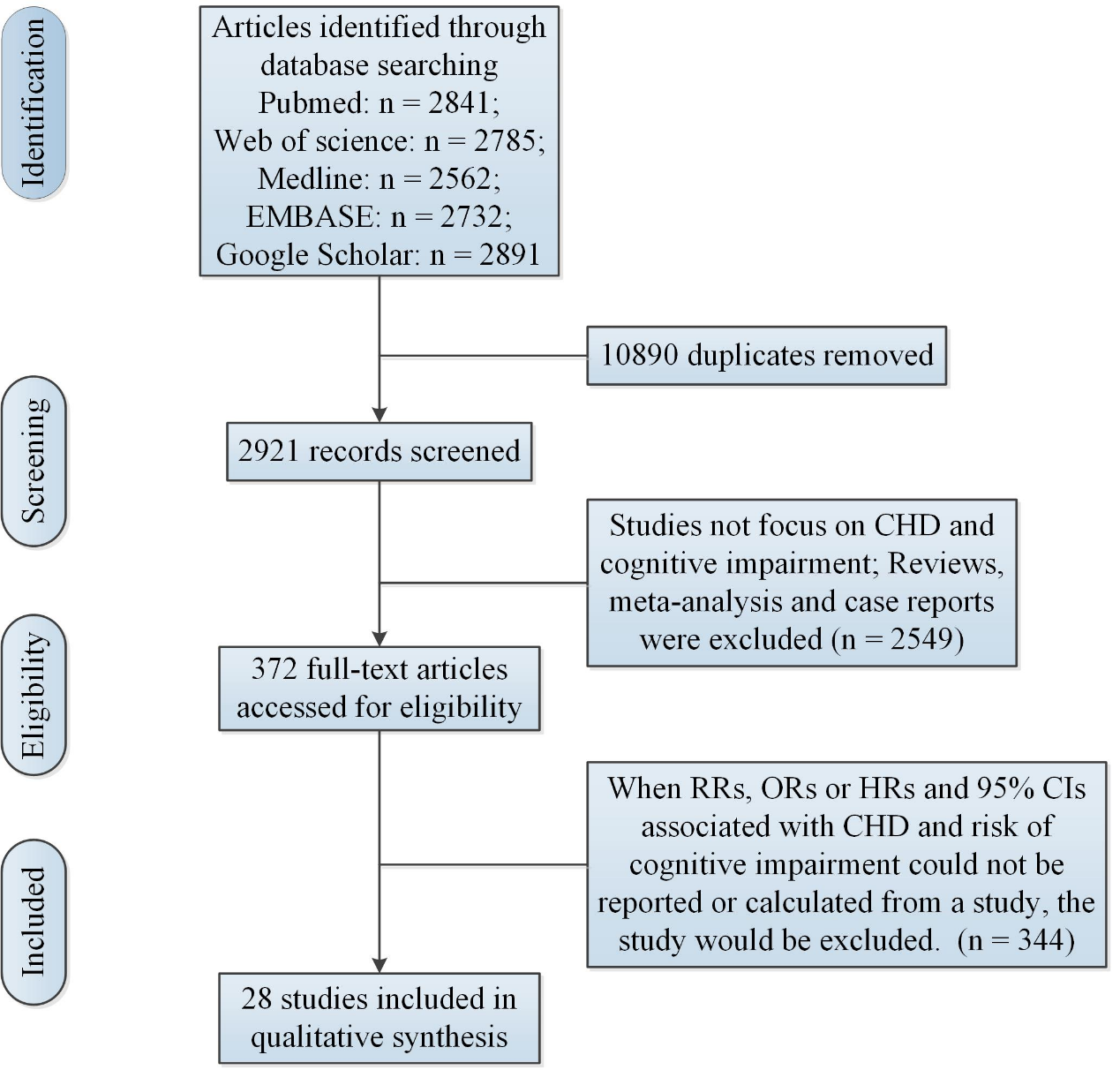


Supplementary figure 1. Flow of information through the different phases of a meta-analysis.

Supplement: Supplementary file 1 — Fig S1 [file BRB3-11-e02108-s002.docx]
