# Supplementary material for: Associations between coronary heart disease and risk of cognitive impairment: A meta‐analysis
Source: Brain Behav. 2021 Mar 20;11(5):e02108. doi: 10.1002/brb3.2108 (PMC8119850; doi:10.1002/brb3.2108)
Supplement: Supplementary file 3 — Fig S3 [file BRB3-11-e02108-s003.docx]

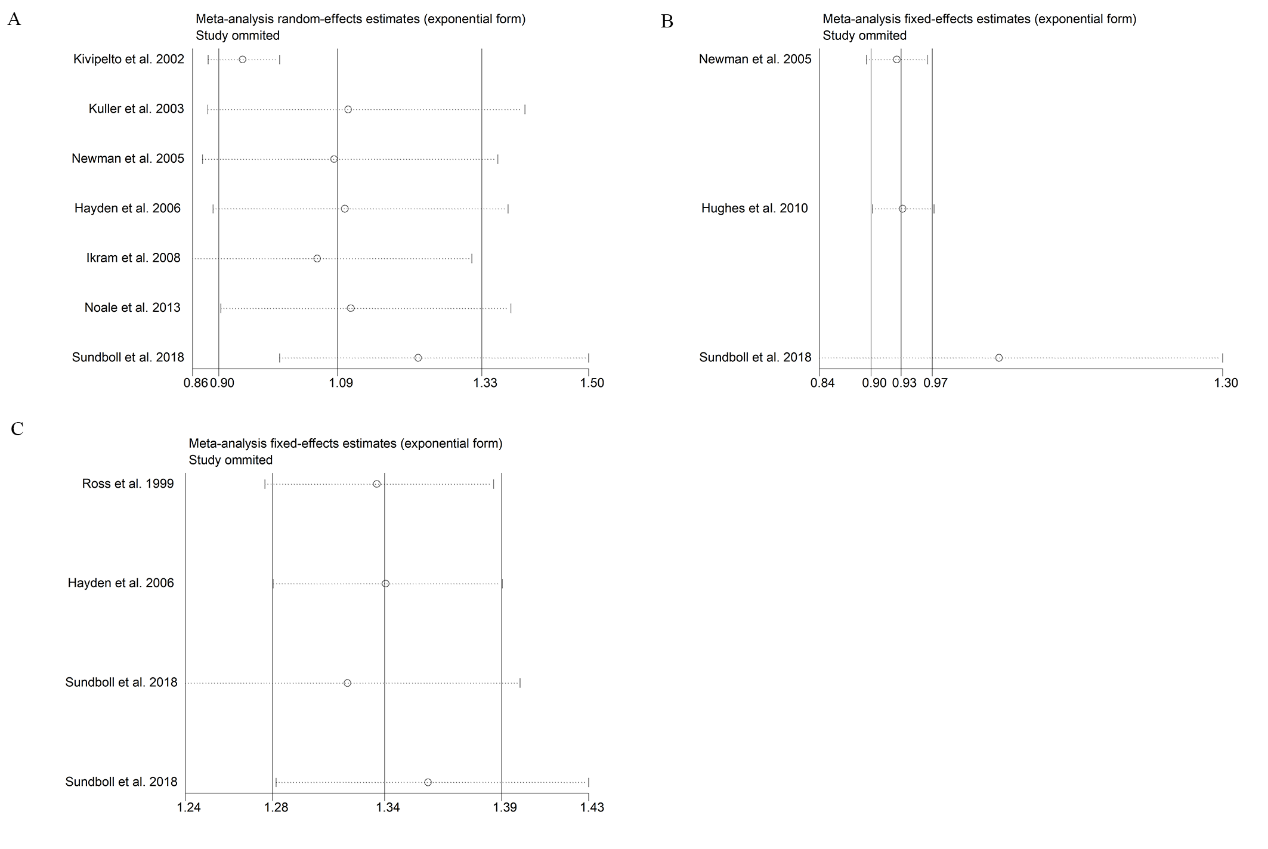


Supplementary figure 3. Sensitivity analyses regarding associations between MI and AD (A), AP and AD (B), CHD and VD (C). Abbreviations: AD, Alzheimer's disease; AP, angina pectoris; CHD, coronary heart disease; MI, myocardial infarction; VD, vascular dementia.
