# Supplementary material for: Associations between coronary heart disease and risk of cognitive impairment: A meta‐analysis
Source: Brain Behav. 2021 Mar 20;11(5):e02108. doi: 10.1002/brb3.2108 (PMC8119850; doi:10.1002/brb3.2108)
Supplement: Supplementary file 4 — Fig S4 [file BRB3-11-e02108-s001.docx]

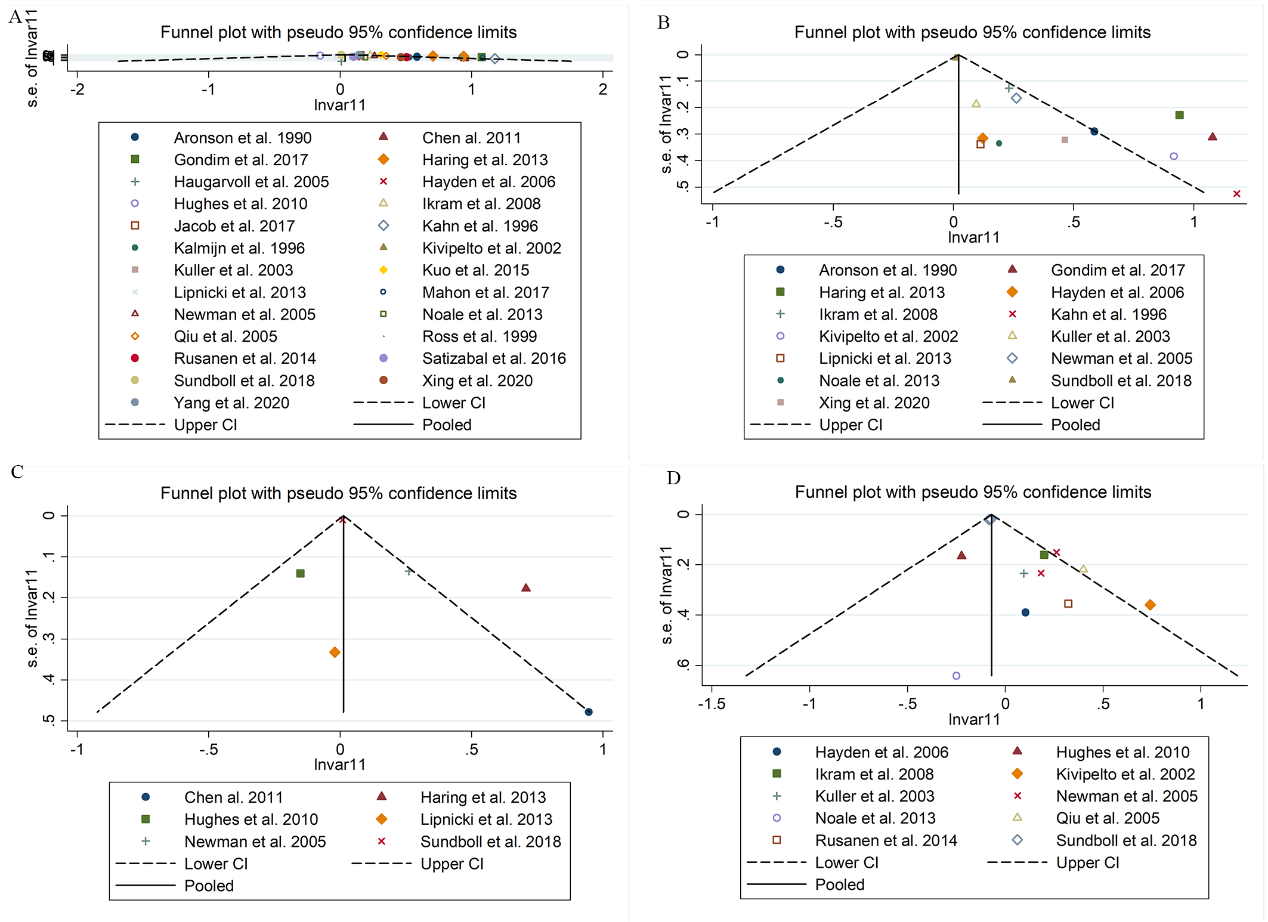


Supplementary figure 4. Funnel graphs regarding associations between CHD and risk of all-cause cognitive impairment (A), MI and risk of all-cause cognitive impairment (B), AP and risk of all-cause cognitive impairment (C), CHD and AD (D). Abbreviations: AD, Alzheimer's disease; AP, angina pectoris; CHD, coronary heart disease; MI, myocardial infarction.
