# Supplementary material for: Associations between coronary heart disease and risk of cognitive impairment: A meta‐analysis
Source: Brain Behav. 2021 Mar 20;11(5):e02108. doi: 10.1002/brb3.2108 (PMC8119850; doi:10.1002/brb3.2108)
Supplement: Supplementary file 6 — Table S1 [file BRB3-11-e02108-s004.docx]

Supplementary table 1. Characteristics of included studies regarding association between coronary heart disease and risk of cognitive impairment or dementia.

| Study/ Year | Country | Study type | Study cohort | Sample size | Mean age  (years) | Gender  (male%) | CHD type | Median follow-up time | type and cases of cognitive impairment | Adjustment | Result |
| --- | --- | --- | --- | --- | --- | --- | --- | --- | --- | --- | --- |
| Aronson et al. 1990 ^[^[^1^](#_ENREF_1)^]^ | USA | Cohort | Bronx Aging study | 442 | 79.2 | NR | MI | 2-7 years | Dementia: NR | Sex, age, word fluency, Blessed IMC error score | HR: 1.8 (1.03-3.2) |
| Kalmijn et al. 1996 ^[^[^2^](#_ENREF_2)^]^ | Netherlands | Cohort | Zuthpen Elderly study | 353 | 74.6 | NR | CHD | 3 years | Cognitive impairment: NR | Age, education, baseline MMSE score | OR: 1.7 (0.8, 3.5) |
| Kahn et al. 1996 ^[^[^3^](#_ENREF_3)^]^ | USA | Cohort | Bronx Aging Study | 459 | 79 | 35.3% | MI | Max. 10 years | Dementia: NR | Age, gender, Blessed score, serum cholesterol, digoxin use, body mass, index, diabetes, smoking status, prior Ml as determined by history of ECG, hypertension according to history, and cardiomegaly according to the results of chest x-ray (cardiothoracic ratio 2:50%). | RR: 3.25 (1.96-15.35) |
| Ross et al. 1999 ^[^[^4^](#_ENREF_4)^]^ | USA | Cohort | the Honolulu-Asia Aging Study | 2,916 | 71 | NR | CHD | Max. 28  years | VaD: 68 | Age, education, hypertension, diabetes, Western diet  preference, use of Vitamin E, 1-hour postprandial  glucose at examination | OR: 2.5 (1.35, 4.62) |
| Kivipelto et al. 2002 ^[^[^5^](#_ENREF_5)^]^ | Finland | Cohort | the North Karelia Project and the Finnish part of the Multinational Monitoring of Trends and Determinants in Cardiovascular Disease studies | 1287 | NR | 61.8% | MI | 21 years | AD: 48  AD or VD: 52 | Age, apolipoprotein E genotype, education level, sex, smoking status, alcohol consumption | AD: RR: 2.1 (1.1, 4.5);  AD or VD: RR: 2.5 (1.2, 5.4) |
| Kuller et al. 2003 ^[^[^6^](#_ENREF_6)^]^ | USA | Cohort | Cardiovascular Health Study | 3608 | 73 | 40.9% | MI | Max. 8 years | Dementia: 480; AD: 330; VD, alone or with AD: 128 | Age | Dementia: RR: 1.1 (0.75, 1.57); AD: RR: 1.1 (0.70, 1.75); VD, alone or with AD: RR: 1.2 (0.64, 2.34) |
| Solfrizzi et al. 2004 ^[^[^7^](#_ENREF_7)^]^ | Italy | Cohort | Italian Longitudinal Study on Aging, MCI | 2963 | 80.7 (2.5) | 49.6% | CHD | 3.5 years | MCI: 176; dementia: 16 | Age, sex, and educational level | MCI: RR: 1.42 (0.86, 2.27); dementia: RR: 1.71 (0.32, 6.78) |
| Haugarvoll et al. 2005 ^[^[^8^](#_ENREF_8)^]^ | Norway | Cohort | Parkinson Disease | 171 | 69.8 (8.0) | 49.1% | CHD | 4 years | dementia: 43 | NR | RR: 1.01 (0.16, 5.04) |
| Newman et al. 2005 ^[^[^9^](#_ENREF_9)^]^ | USA | Cohort | Cardiovascular Health Study | 2539 | NR | 60.1% | MI, AP | 5.4 years | Dementia: 396;  AD with No  VD: 245 | Age at baseline, race, education, income, apolipoprotein e-4 allele, Modiﬁed Mini-Mental State Examination score at the time of the brain  magnetic resonance scan | MI: Dementia: RR: 1.3 (1.0, 1.9) ; AD: RR: 1.2 (0.8, 2.0);  AP: Dementia:  RR: 1.3 (1.0, 1.7); AD: RR: 1.3 (1.0–1.8) |
| Qiu et al. 2005 ^[^[^10^](#_ENREF_10)^]^ | Sweden | Cohort | Kungsholmen | 1301 | NR | 75.0% | CHD | 6 years | Dementia: 350; AD: 260 | Age，education，APOEepsilon4 allele, cognitive impairment, stroke, atrial fibrillation, antihypertensive drug use | Dementia: RR:1.42 (1.00-2.03);  AD: RR:1.49 (0.97-2.29) |
| Hayden et al. 2006 ^[^[^11^](#_ENREF_11)^]^ | USA | Cohort | Cache County Study | 3264 | 73.7 | 57.7% | MI | 3 years | Dementia: 185; AD: 104; VD: 37 | Age, sex, education, hypertension, high cholesterol, diabetes, obesity, stroke, CABG | Dementia: HR: 1.13 (0.59–2.03); AD: 1.11 (0.49-2.26); VD: 1.06 (0.34–2.93) |
| Ikram et al. 2008 ^[^[^12^](#_ENREF_12)^]^ | Netherlands | cohort | the Rotterdam Study | 6347 | 68.7 | 41.1% | MI | 9.3 years | Dementia: 613;  AD | Age, sex, and additionally adjusted for presence of APOE 4 allele, systolic blood pressure, diastolic blood pressure, body mass index, atrial fibrillation, diabetes mellitus, current smoking, intima media thickness, total cholesterol and high-density lipid cholesterol | Dementia: HR: 1.26 (0.98, 1.61); AD: HR: 1.22 (0.89, 1.67) |
| Hughes et al. 2010 ^[^[^13^](#_ENREF_13)^]^ | Sweden | Case-control | the Swedish Twin Registry | 3664 | 48.2 | 62.0% | AP | 31 years | Dementia: 355; AD: 240 | Age, gender, education, smoking, alcohol drinking, BMI, total food compared to others, marital status, exercise | RR: dementia: 0.86 (0.65-1.13); AD: 0.80 (0.58-1.11) |
| Li et al. 2011 ^[^[^14^](#_ENREF_14)^]^ | China | cohort | MCI | 837 | NR | 41.6% | MI | 5 years | AD: 298 | Age, sex, education, occupation, depressive symptoms, APOE4, baseline MMSE, and ADL score | HR:1.051 (0.670–1.648) |
| Chen al. 2011 ^[^[^15^](#_ENREF_15)^]^ | United Kingdom | cohort | Anhui cohort study | 1,307 | ≥65 | NR | AP | 7.5 years | dementia | Age, sex, education, main occupation, annual income, urban rurality, BMI, smoking habits, hobby's, relationship with others, living with others, worrying, hypochondriasis, anything severely upsetting, horrifying experience | OR: 2.58, 1.01–6.59 |
| Haring et al. 2013 ^[^[^16^](#_ENREF_16)^]^ | USA | cohort | the Women’s Health Initiative Memory Study | 6455 | 65 to 79 | 0% | MI  AP | 8.4 years | MI: 294; AP: 291 | age, education, race, HTR arm, baseline 3MSE, alcohol intake, smoking status, physical activity, diabetes status, sleep hours, hypertension status, BMI, depression, waist-hip ratio, hypercholesterolemia, and aspirin use | MCI: MI: RR: 2.56 (1.64, 4.01); AP: RR: 2.03 (1.43, 2.87) |
| Lipnicki et al. 2013 ^[^[^17^](#_ENREF_17)^]^ | Australia | cohort | the Sydney Memory and Ageing Study | 889 | 78.6 (4.8) | 45.9% | MI  AP  CHD | 2.0 years | MCI: 77; dementia: 16  CI | Age, sex | MI : OR: 1.12 (0.58, 2.19); AP:  OR: 0.98 (0.51, 1.88); CHD: OR: 0.97 (0.55,  1.71) |
| Noale et al. 2013 ^[^[^18^](#_ENREF_18)^]^ | Italy | cohort | the Italian Longitudinal Study on Aging | 2501 | 71.3 (5.3) | 43.7% | MI | 7.8 years | Dementia: 194; AD: 83; VD: 50; other dementia or mixed dementia: 61 | NR | Dementia: RR: 1.21 (0.63, 2.34); AD: RR: 0.78 (0.22, 2.71) |
| Rusanen et al. 2014 ^[^[^19^](#_ENREF_19)^]^ | Finland | cohort | the Cardiovascular Risk Factors, Aging and Dementia(CAIDE)study | 738 | 68 | 37.6% | CAD | 7.8 years | Dementia: 127; AD: 102 | Gender, education, midlife systolic blood pressure, cholesterol, body mass index, APOE, midlife smoking, physical activity, diabetes or impaired glucose tolerance and stroke at late-life | Dementia: RR: 1.66 (0.87, 3.16); AD: RR: 1.38 (0.69, 2.77) |
| Kuo et al. 2015 ^[^[^20^](#_ENREF_20)^]^ | China Taiwan | cohort | Taiwan National Health Insurance | 67,066 | 62.1 (11.4) | 48.4% | CAD | 11 years | NR | NR | Dementia: HR: 1.37 (1.25, 1.50) |
| Nesteruk et al. 2015 ^[^[^21^](#_ENREF_21)^]^ | Poland | cohort | MCI | 101 | 62.7 | 42.6% | MI | 2 years | Dementia: 17 | NR | Dementia: HR: 4.82 |
| Satizabal et al. 2016 ^[^[^22^](#_ENREF_22)^]^ | USA | cohort | Framingham | 2090 | 72 (9) | 44% | CHD | Max. 5 years | NR | NR | Dementia: HR: 1.11 (0.65, 1.88) |
| Jacob et al. 2017 ^[^[^23^](#_ENREF_23)^]^ | Germany | Case-control | The Disease Analyzer database (IMS Health) | 3,604 MCI patients and 3,604 controls | 75.2 (9.1) | 45.3% | CHD | NA | MCI: 1031 | NR | OR: 1.17 (1.04, 1.32) |
| Gondim et al. 2017 ^[^[^24^](#_ENREF_24)^]^ | Brazil | Cross-sectional | Brazilian community-dwelling older adults | 461 | 60- 69 | 27.99% | MI | NA | FCI: NR | potential confounders | OR: 2.94 (1.59 to 5.42) |
| Mahon et al. 2017 ^[^[^25^](#_ENREF_25)^]^ | New Zealand | cohort | Post-stroke, the fourth Auckland Stroke Re-  gional Outcomes Study | 257 | 67.93 ± 13.59 | 53% | CHD | 4 years | CHD: NR | all variables identified as statistically significant predictors from the univariate analysis | OR 2.96, 95% CI 1.35–6.49 |
| Sundbøll et al. 2018 ^[^[^26^](#_ENREF_26)^]^ | Denmark | cohort | Danish registry | 1,213,517 | 67 | 65.9 | MI  AP | 7.7–9.8 years | MI: Dementia: 11,3, 34; AD:  3615; VD: 2092; other dementia: 5627 | NR | MI: Dementia: HR: 1.01, 0.98–1.03; AD: 0.92, 0.88–0.95; VD: 1.35, 1.28–1.43;  other dementia: 0.98, 0.95–1.01;  AP:  Dementia: HR: 1.01 (0.99–1.03); AD: 0.93 (0.89–0.96); VD: 1.31 (1.23–1.39);  other dementia: 0.98 (0.95–1.01) |
| Yang et al. 2020 ^[^[^27^](#_ENREF_27)^]^ | UK | cohort | stroke patients | 63,959 | 75 (64–83) | 50.9% | CHD | 3.6 years | Dementia: 7,265 | demographics and lifestyle | HR: 1.16  (1.10–1.23) |
| Xing et al. 2020 ^[^[^28^](#_ENREF_28)^]^ | China | Case-control | medical care or routine physicals at the Geriatric Medicine Department of the Beijing Friendship Hos-  pital | 614 | 82 ± 7 | 68% | MI | NA | cognitive impairment | NR | OR: 1.59 (0.85, 3.00) |

Abbreviations: AD, Alzheimer’s disease; ADL, activities of daily living; AP, angina pectoris; APOE, apolipoprotein E; BMI, body mass index; CABG, coronary artery bypass graft; CAD, coronary artery disease; CHD, coronary heart disease; ECG, electrocardiogram; FCI, functional cognitive impairment; HR, hazard ratio; IHD, ischemic heart disease; IMC, information-memory-concentration; MCI, mild cognitive impairment; MI, myocardial infraction; MMSE, Mini-Mental State Examination; NA, not applicable; NR, not reported; OR, odds ratio; RR, relative risk; USA, united states; VD, vascular dementia.
