# Supplementary material for: Associations between coronary heart disease and risk of cognitive impairment: A meta‐analysis
Source: Brain Behav. 2021 Mar 20;11(5):e02108. doi: 10.1002/brb3.2108 (PMC8119850; doi:10.1002/brb3.2108)
Supplement: Supplementary file 7 — Supplementary Material [file BRB3-11-e02108-s006.docx]

Supplementary references

[1] ARONSON M K, OOI W L, MORGENSTERN H, et al. Women, myocardial infarction, and dementia in the very old [J]. Neurology, 1990, 40(7): 1102-6.

[2] KALMIJN S, FESKENS E J, LAUNER L J, et al. Cerebrovascular disease, the apolipoprotein e4 allele, and cognitive decline in a community-based study of elderly men [J]. Stroke, 1996, 27(12): 2230-5.

[3] KAHN S, FRISHMAN W H, WEISSMAN S, et al. Left ventricular hypertrophy on electrocardiogram: prognostic implications from a 10-year cohort study of older subjects: a report from the Bronx Longitudinal Aging Study [J]. Journal of the American Geriatrics Society, 1996, 44(5): 524-9.

[4] ROSS G W, PETROVITCH H, WHITE L R, et al. Characterization of risk factors for vascular dementia: the Honolulu-Asia Aging Study [J]. Neurology, 1999, 53(2): 337-43.

[5] KIVIPELTO M, HELKALA E L, LAAKSO M P, et al. Apolipoprotein E epsilon4 allele, elevated midlife total cholesterol level, and high midlife systolic blood pressure are independent risk factors for late-life Alzheimer disease [J]. Annals of internal medicine, 2002, 137(3): 149-55.

[6] KULLER L H, LOPEZ O L, NEWMAN A, et al. Risk factors for dementia in the cardiovascular health cognition study [J]. Neuroepidemiology, 2003, 22(1): 13-22.

[7] SOLFRIZZI V, PANZA F, COLACICCO A M, et al. Vascular risk factors, incidence of MCI, and rates of progression to dementia [J]. Neurology, 2004, 63(10): 1882-91.

[8] HAUGARVOLL K, AARSLAND D, WENTZEL-LARSEN T, et al. The influence of cerebrovascular risk factors on incident dementia in patients with Parkinson's disease [J]. Acta neurologica Scandinavica, 2005, 112(6): 386-90.

[9] NEWMAN A B, FITZPATRICK A L, LOPEZ O, et al. Dementia and Alzheimer's disease incidence in relationship to cardiovascular disease in the Cardiovascular Health Study cohort [J]. Journal of the American Geriatrics Society, 2005, 53(7): 1101-7.

[10] QIU C X, WINBLAD B, FRATIGLIONI L. [Risk factors for dementia and Alzheimer' s disease-findings from a community-based cohort study in Stockholm, Sweden] [J]. Zhonghua liu xing bing xue za zhi = Zhonghua liuxingbingxue zazhi, 2005, 26(11): 882-7.

[11] HAYDEN K M, ZANDI P P, LYKETSOS C G, et al. Vascular risk factors for incident Alzheimer disease and vascular dementia: the Cache County study [J]. Alzheimer disease and associated disorders, 2006, 20(2): 93-100.

[12] IKRAM M A, VAN OIJEN M, DE JONG F J, et al. Unrecognized myocardial infarction in relation to risk of dementia and cerebral small vessel disease [J]. Stroke, 2008, 39(5): 1421-6.

[13] HUGHES T F, ANDEL R, SMALL B J, et al. Midlife fruit and vegetable consumption and risk of dementia in later life in Swedish twins [J]. The American journal of geriatric psychiatry : official journal of the American Association for Geriatric Psychiatry, 2010, 18(5): 413-20.

[14] LI J, WANG Y J, ZHANG M, et al. Vascular risk factors promote conversion from mild cognitive impairment to Alzheimer disease [J]. Neurology, 2011, 76(17): 1485-91.

[15] CHEN R, HU Z, WEI L, et al. Incident dementia in a defined older Chinese population [J]. PloS one, 2011, 6(9): e24817.

[16] HARING B, LENG X, ROBINSON J, et al. Cardiovascular disease and cognitive decline in postmenopausal women: results from the Women's Health Initiative Memory Study [J]. Journal of the American Heart Association, 2013, 2(6): e000369.

[17] LIPNICKI D M, SACHDEV P S, CRAWFORD J, et al. Risk factors for late-life cognitive decline and variation with age and sex in the Sydney Memory and Ageing Study [J]. PloS one, 2013, 8(6): e65841.

[18] NOALE M, LIMONGI F, ZAMBON S, et al. Incidence of dementia: evidence for an effect modification by gender. The ILSA Study [J]. International psychogeriatrics, 2013, 25(11): 1867-76.

[19] RUSANEN M, KIVIPELTO M, LEV LAHTI E, et al. Heart diseases and long-term risk of dementia and Alzheimer's disease: a population-based CAIDE study [J]. Journal of Alzheimer's disease : JAD, 2014, 42(1): 183-91.

[20] KUO S C, LAI S W, HUNG H C, et al. Association between comorbidities and dementia in diabetes mellitus patients: population-based retrospective cohort study [J]. Journal of diabetes and its complications, 2015, 29(8): 1071-6.

[21] RICOTTI V, MANDY W P, SCOTO M, et al. Neurodevelopmental, emotional, and behavioural problems in Duchenne muscular dystrophy in relation to underlying dystrophin gene mutations [J]. Developmental medicine and child neurology, 2016, 58(1): 77-84.

[22] SATIZABAL C, BEISER A S, SESHADRI S. Incidence of Dementia over Three Decades in the Framingham Heart Study [J]. The New England journal of medicine, 2016, 375(1): 93-4.

[23] JACOB L, BOHLKEN J, KOSTEV K. Risk Factors for Mild Cognitive Impairment in German Primary Care Practices [J]. Journal of Alzheimer's disease : JAD, 2017, 56(1): 379-84.

[24] GONDIM A S, COELHO FILHO J M, CAVALCANTI A A, et al. Prevalence of functional cognitive impairment and associated factors in Brazilian community-dwelling older adults [J]. Dementia & neuropsychologia, 2017, 11(1): 32-9.

[25] MAHON S, PARMAR P, BARKER-COLLO S, et al. Determinants, Prevalence, and Trajectory of Long-Term Post-Stroke Cognitive Impairment: Results from a 4-Year Follow-Up of the ARCOS-IV Study [J]. Neuroepidemiology, 2017, 49(3-4): 129-34.

[26] SUNDB LL J, HORV TH-PUH E, ADELBORG K, et al. Higher Risk of Vascular Dementia in Myocardial Infarction Survivors [J]. Circulation, 2018, 137(6): 567-77.

[27] YANG Z, EDWARDS D, BURGESS S, et al. Association of Prior Atherosclerotic Cardiovascular Disease with Dementia After Stroke: A Retrospective Cohort Study [J]. Journal of Alzheimer's disease : JAD, 2020, 77(3): 1157-67.

[28] XING Y L, CHEN M A, SUN Y, et al. Atherosclerosis, its risk factors, and cognitive impairment in older adults [J]. Journal of geriatric cardiology : JGC, 2020, 17(7): 434-40.
